# Geographic variations and temporal trends in prostate cancer in Martinique over a 25-year period

**DOI:** 10.1186/1756-0500-7-262

**Published:** 2014-04-23

**Authors:** Moustapha Dieye, Rishika Banydeen, Jonathan Macni, Stephane Michel, Jacqueline Veronique-Baudin, Annie Sasco, Patrick Escarmant, Clarisse Joachim

**Affiliations:** 1 Pôle de Cancérologie Hématologie Urologie, Registre des Cancers de la Martinique – UF 1441 Recherche & Registre, Centre Hospitalier Universitaire de la Martinique, 127 Route de Redoute, Fort-de-France 97200, Martinique; 2 Registre des Cancers de la Martinique, AMREC, 127 Route de Redoute, Fort-de-France, Martinique; 3 AMREC, 127 Route de Redoute, Fort-de-France, Martinique; 4 Université Bordeaux, ISPED, Centre INSERM U897-Epidémiologie-Biostatistique, Bordeaux F-33000, France; 5 INSERM, ISPED, Centre INSERM U897- Epidémiologie-Biostatistique, Bordeaux F-33000, France; 6 Pôle de Cancérologie Hématologie Urologie, Centre Hospitalier Universitaire de la Martinique, Fort-de-France BP 632, 97261, Martinique

**Keywords:** Prostate cancer, Incidence rate, Age-period-cohort model, Period analysis, Spatial distribution, Environmental exposure

## Abstract

**Background:**

In Martinique, prostate cancer incidence rates have been increasing since the 1980s and are actually among the highest worldwide. Exposure to lifestyle (changes in dietary habits), environmental factors (exposure to organochlorine pesticides) and modifications in diagnostic and screening procedures, are favored etiological hypotheses. The aim of the present study is to describe and interpret prostate cancer incidence trends over the past 25 years (1981–2005) in Martinique.

**Methods:**

Data on incident prostate cancer cases from 1981 to 2005 were obtained from the population-based Martinique Cancer Registry. World age-standardised incidence rates were calculated and an age-period-cohort model was used to determine average annual variations for prostate cancer during the study period. Age and period effects were assessed, employing the method proposed by Clayton and Schifflers. Relative changes in prostate cancer incidence, at five-year intervals between 1981 and 2005, were also studied with an organochlorine pesticide exposure index, built as a proxy of the relative intensity of chlordecone use on the island between 1973 and 1993.

**Results:**

Prostate cancer incidence was found to increase by 5.07% annually between 1981 and 2005. Compared to 1981–1985, prostate cancer relative risk, in men aged 50–74 years and 75 years and above was respectively 5.98% and 3.07% from 2001 to 2005. An inverse association between population pesticide exposure levels and prostate cancer risk was also highlighted, with highest prostate cancer incidences observed in urban zones showing the lowest soil contamination levels by the chlordecone pesticide (zone 1).

**Conclusion:**

No conclusive association was found between the intensity of pesticide use and the subsequent rise in prostate cancer incidence. However, it remains necessary to develop and reinforce continuous monitoring of prostate cancer incidence and mortality trends on the island. Further studies are also needed in order to consider other risk factors such as modifications in diagnostic and screening procedures over the last 25 years.

## Background

The increasing burden of cancer is becoming more and more of a public health concern in the French department of Martinique and is the leading cause of death in men and women. Over the last decade, the incidences of several tumors (prostate, breast, colorectal, non-Hodgkin’s lymphoma…) have been on the rise in the general population.

Changes in life expectancy and lifestyle habits, as well as improved diagnostic procedures such as the implementation of screening techniques (mammography, Prostate Specific Antigen and hemoccult tests) have been put forward to explain these time trends [1].

Prostate cancer incidence in Martinique is today one of the highest worldwide [1,2].

In recent years, several studies have aroused public and scientific concern about the health effects of pesticides. In spite of many publications documenting these effects, in particular the carcinogenicity of pesticides, there remains deep controversy over this issue. While acute effects of pesticides have been well described in literature, especially with respect to organochlorine poisoning, chronic effects of pesticide exposure have been much more difficult to assess [3].

However, while the natural environment of the French department is widely recognized as being contaminated by the substantial use of pesticides due to intensive banana farming activities [4,5], their role in prostate cancer etiology has not yet been clearly defined and requires thorough investigation. Particular questioning remains about chlordecone- mediated pollution. Chlordecone (also known as Kepone) is an organochlorine insecticide, extensively used in Martinique and the rest of the French West Indies, to control the banana root borer from 1973 to 1993. The pesticide’s properties (estrogenicity, high stability, high persistence in the environment) has engendered the widespread permanent contamination of soils, river water, wild animals, and vegetables growing on polluted soils. The latter constitute the primary source of foodstuff contamination, and hence remain an indirect source of exposure for local inhabitants [4-9].

The aim of the present study is to describe and interprete prostate cancer incidence trends in Martinique from 1981 to 2005.

## Methods

Data on incident prostate cancer cases from 1981 to 2005 were obtained from the population-based Martinique Cancer Registry. The Registry is a full member of the International Association of Cancer Registries (IACR). Data collection and coding are conducted as recommended by the IARC [10].

Registered male cases corresponding to the code C61 (excluding sarcomas, leukaemia, lymphomas and melanomas) of the International Classification of Diseases for Oncology [ICD-O-3] were included. Patient identification number, date of birth, cancer diagnosis date, histological code and residential postal code at diagnosis were collected. Data quality was assessed on the basis of recommendations from the IARC [10]. Each cancer case was confirmed, on average, by at least two sources (medical oncology services and private pathology laboratories). Ninety-five percent of tumors had a microscopic confirmation at diagnosis. We furthermore evaluated exhaustiveness using the two main data sources by using “capture-recapture” methods [11]. With case notifications cross-checked by the use of a wide range of information sources, the exhaustiveness of the cancer registry was estimated at 97.5%.

Demographic information was collected from the French National Institute of Statistics and Economic studies (INSEE), which provides inter-census estimates of population sizes, as well as data on age and sex distributions for the population of Martinique.

A pesticide exposure index was built by the French Regional Agency for Industry, Research and Environment (DIREN) to assess cumulative population exposure to the chlordecone pesticide. This index was built using the percentage of potentially polluted ground surfaces as a proxy of the relative intensity of chlordecone use from 1973 to 1993 [12] with four levels of risk of exposure for the population: none, low, medium and high.

The different municipalities of the island were then categorized into four zones, according to the pollution levels by chlodecone: less than 10 percent of chlordecone- contaminated ground (zone 1), between 10 and 20 percent (zone 2), between 20 and 30 percent (zone 3) and more than 30 percent of soil contamination by chlordecone (zone 4). Areas with high chlordecone contamination levels (zones 3 and 4) are essentially agricultural rural areas, while areas with low chlordecone contamination levels (zones 1 and 2) are essentially urban countries.

World age-standardized incidence rates were calculated according to 5-year period intervals. A first trend analysis of trends in prostate cancer incidence between 1981 and 2005 was conducted using an age-period-cohort model to determine average annual variations (average annual percent change). Age and period effects were assessed, employing the method of Clayton and Schifflers [13,14], with two log-linear Poisson regression models, fitted separately for (1) age alone and then (2) age and period (age-drift model). A ‘drift’ parameter, an incidence trend which can be interpreted as a period effect, was also computed.

In a second analysis, relative changes in prostate cancer incidence, in men aged 50 to 74 years and 75 years and above, were calculated for the 1981–2005 period. Analysis was stratified according to 5-year period intervals, using the 1981–1985 period as a reference category. Statistical comparisons of relative prostate cancer risk were also made by assuming that the observed number of prostate cancer events followed a Poisson distribution. Relative changes in prostate cancer incidence were analyzed according to diagnosis period and chlordecone exposure level, using a Poisson Regression model. For all analyses, prostate cancer cases with missing residential postal codes at diagnosis were excluded. Significance tests and parameter estimations were conducted using the maximum likelihood method. All analyses were carried out using SAS 9.2 software [SAS Institute].

## Results

Between 1981 and 2005, there were 6097 cases of prostate cancer registered at the Martinique Cancer Registry. We excluded 404 cases (6.6%) because of missing postal codes. In the end, 5693 prostate cancer cases were included in the study.

The number of prostate cancer cases according to 5-year period intervals, varied from 252 to 2281 newly-diagnosed cases over the study period. Table 1 details the age-standardised prostate cancer incidence rates which increased from 36.9 per 100 000 men-years (period 1981–1985) to 164 per 100 000 men-years (2001–2005).

**Table 1:** Prostate cancer incidence from 1981 to 2005 in Martinique

**Period**	**Number of cases**	**Crude incidence rate per 100000 men-years [95% CI]**	**Age-standardised incidence rate**^**† **^per 100000 men-years [95% CI]^*^
1981-1985	252	40.3 [35.4, 45.3]	36.9 [32.3, 41.5]
1986-1990	602	71.5 [65.8, 77.2]	59.7 [54.8, 64.6]
1991-1995	964	109 [103, 116]	81.7 [76.3, 87.0]
1996-2000	1594	177 [168, 186]	123 [117, 130]
2001-2005	2281	248 [238, 258]	164 [157, 171]

^*^95% CI: 95% Confidence Interval.

^*†*^*World population of 1960 used as standard.*

### Incidence variations according to age

Prostate cancer relative risk, adjusted by age, from 1981 to 2005 (with 1981–1985 as reference period), is presented in Table 2 for the age groups 50–74 years and 75 years and above. From 1981 to 2005, relative rates for the disease increased more rapidly in the 50–74 year age group as compared to 75 year-olds and above. It was only since 1996 that relative incidence rates for 75 year-olds and above approximated values registered for 50–74 year olds a decade earlier (1991).

**Table 2:** Relative changes in prostate cancer incidence from 1981 to 2005 (reference period: 1981–1985)

**Age (years)**	**Period**	RR^*^	**[95% CI]**^**†**^		**p-value**^**[**^
50-74	1981-1985	1.00	-	-	-.
	1986-1990	1.86	1.53	2.27	<.0001
	1991-1995	2.63	2.18	3.18	<.0001
	1996-2000	4.38	3.67	5.24	<.0001
	2001-2005	5.98	5.02	7.13	<.0001
> 75	1981-1985	1.00	-	-	-.
	1986-1990	1.62	1.28	2.06	0.0003
	1991-1995	2.21	1.77	2.77	<.0001
	1996-2000	2.65	2.13	3.29	<.0001
	2001-2005	3.07	2.48	3.79	<.0001

^*^RR: Relative risk;^†^95% CI*: 95% Confidence Interval;*^*[*^*level of significance: p-value < 5%*.

### Incidence variations according to “pesticide exposure”

Relative incidence rates for prostate cancer, adjusted by age and population pesticide exposure levels between 1981 and 2005, are presented in Table 3. In zone 1 (no population exposure to chlordecone), relative rates are found to be statistically significant and increasing from 1981 to 2005 (p < 0.05 for each study period). As for the other zones, relative prostate cancer risk was not statistically significant, except for zone 3 where significant changes in prostate cancer risk were observed between 1996 and 2005.

**Table 3:** Relative changes in age-standardised incidence for prostate cancer, by level of population exposure to chlordecone, from 1981 to 2005 (reference period: 1981–1985)

Zones^*^	**Period**	**RR**^**†**^	**95% CI**^**[**^		**p-value**^**§**^
1	1981-1985	1.00	-	-	-.
	1986-1990	1.32	1.05	1.67	0.0189
	1991-1995	1.72	1.38	2.14	<.0001
	1996-2000	2.10	1.69	2.60	<.0001
	2001-2005	2.35	1.90	2.91	<.0001
2	1981-1985	1.00	-	-	-.
	1986-1990	0.86	0.63	1.16	0.3174
	1991-1995	0.94	0.70	1.24	0.6512
	1996-2000	1.01	0.77	1.32	0.9528
	2001-2005	1.19	0.91	1.55	0.1960
3	1981-1985	1.00	-	-	-.
	1986-1990	0.89	0.65	1.24	0.5115
	1991-1995	1.04	0.77	1.42	0.7801
	1996-2000	1.38	1.04	1.84	0.0272
	2001-2005	1.59	1.17	2.04	0.0024
4	1981-1985	1.00	-	-	-.
	1986-1990	0.84	0.58	1.22	0.3551
	1991-1995	1.16	0.82	1.64	0.3891
	1996-2000	1.36	0.97	1.89	0.0696
	2001-2005	1.37	0.99	1.89	0.0563

^*^Zone 1: <10% chlordecone contaminated ground: no population exposure to chlordecone; Zone 2: 10-20% chlordecone contaminated ground: low population exposure to chlordecone; Zone 3: 20-30% chlordecone contaminated ground: medium population exposure to chlordecone; Zone 4: >30% chlordecone contaminated ground: high population exposure to chlordecone;^†^*RR: Relative risk;*^[^95% CI*: 95% Confidence Interval;*^**§**^*level of significance: p-value < 5%*.

## Discussion

Using data from the Martinique Cancer Registry, we examined temporal changes in prostate cancer incidence rates over a 25-year period. During this interval, age- standardized incidence rates (ASR) for prostate cancer increased steadily, with an average rise of 5.07% each year.

On the island, observed incidence variations for this cancer are slightly lower than observed trends in mainland France [15], where the average annual increase for prostate cancer was of 5.3% between 1980 and 2005, with ASRs going from 26 new cases/100 000 men-years in 1980 to 121 new cases/100 000 men-years in 2005. We also found that prostate cancer incidence variations in Martinique differed according to age, with relative rates for the disease increasing more rapidly in 50 to 74 year olds as compared to 75 year-olds and above.

Since the 1960s, there has been a gradual increase in the incidence of prostate cancer in many countries. Reasons for these observed international trends are factors such as population ageing, early diagnosis, introduction of more sensitive diagnostic procedures, availability of prostate specific antigen screening during the early to mid-1990s [16,17], and completeness of reporting [16-18].

Moreover, an inverse association between population pesticide exposure level and prostate cancer risk has been highlighted by our results, with highest prostate cancer incidences observed in zones showing the lowest soil contamination levels by the chlordecone pesticide (zone 1).

Although differences in incidence for zone 3 were statistically significant during the periods 1996–2000 and 2001–2005, the magnitude of these differences tends to be small comparatively to zone 1.

Other arguments exist against a “pesticide effect” in terms of confounding factors such as population density, socioeconomic status and differential access to health care. We can also observe that clinical prostate cancer frequently occurs many years after the initial exposure period [17-19]. In our study, it can thus be imagined that if the organochlorine pesticide chlordecone was mainly used during the 1970s-1990s [4,5], a substantial increase in the number of new prostate cancer cases would have been reported as from 2000. It can therefore be supposed that elapsed time since initial pesticide exposure period (1973–1993) may be insufficient to result in measurable changes in prostate incidence.

A second factor which could explain prostate cancer patterns on the island is the effect of modifications in diagnostic procedures. The noted variation in prostate cancer incidence is higher in 50 to 74 year-olds, who are the most frequently targeted by early diagnosis and screening [20]. Our study also showed a significant period effect (5.07%) which reflects changes in diagnostic activities such as PSA testing, as well as improved registration practices. Indeed, implementations of PSA testing in the 1990s may have influenced incidence trends for prostate cancer on the island.

A few limitations are however to be noted in our study. The first one is regarding the pesticide indicator used to determine population exposure levels to chlordecone. Ground contamination represents only one exposure pathway to pesticides. Exposure routes can be very variable due to the diversity of products used, and changing professional practices (use of protective material, application methods…). Results from the Escal study, targeting the health and eating habits of the population of Martinique, have however tended to confirm the pertinence of the indicator used in our study. This is based on the hypothesis that in zones with high chlordecone contamination levels (essentially agricultural rural areas), residents’ exposure levels were higher, compared to other urban regions because of a higher contamination through the food chain [21].

A second drawback is the fact that we did not take into account certain common risk factors which may have contributed partly to the progression of prostate cancer rates in Martinique. The role of genetic factors, hormonal status and other environmental factors (dietary, infectious) should not be downplayed [22-24]. While aging, ethnicity and a family history of prostate cancer remain the only well-established risk factors for the disease, environment and lifestyle related factors are also suspected. On the island, changes in dietary habits and exposure to organochlorine pesticides are also etiological hypotheses, in addition to genetics and African ancestry [22,25].

The Karuprostate case–control study in Guadeloupe) aimed to identify and to characterize genetic and environmental determinants of prostate cancer onset and evolution in the French West Indies. One of the specific objectives was to test whether chlordecone exposure during adulthood, over a 30-year period, favored the development of prostate cancer [26]. The study’s findings supported the hypothesis that exposure to environmental estrogens increased the risk of prostate cancer and suggested that this association may be affected by genetic background, together with environmental agents related to diet or lifestyle [27].

For the geographical analysis of prostate cancer incidence rates according to pesticide exposure, we used residential postal codes at the time of diagnosis. Interpretation biases, linked to the absence of evaluation of the residential stability of cancer cases from the supposed time of exposure (1973–1993) to the period of diagnosis (1981–2005), might also have been introduced in our study. Moreover, a selection bias is to be supposed as excluded cases, due to missing postal codes, were older and from the earlier diagnostic groups.

## Conclusions

In summary, incidence rates for prostate cancer have been increasing, in Martinique, over the 25-year study period at an annual progression rate of 5.07%, reflecting a period effect. No conclusive association was found between the intensity of chlordecone use and the subsequent rise in prostate cancer incidence. However, it remains necessary to develop and reinforce continuous monitoring of prostate cancer incidence and mortality trends on the island. Furthermore, well-designed and well-executed population-based interdisciplinary studies should help elucidate the independent and combined effects of environmental and genetic factors, as well as modifications in diagnostic and screening practice in prostate cancer etiology.

## Competing interests

The authors declare that they have no competing interests.

## Authors’ contribution

All authors contributed to the development of the analytical plan. All authors read and approved the final manuscript.
